# Reducing Healing Period with DDM/rhBMP-2 Grafting for Early Loading in Dental Implant Surgery

**DOI:** 10.1007/s13770-024-00689-3

**Published:** 2025-01-18

**Authors:** Jeong-Kui Ku, Jung-Hoon Lim, Jung-Ah Lim, In-Woong Um, Yu-Mi Kim, Pil-Young Yun

**Affiliations:** 1 Department of Oral and Maxillofacial Surgery, Section of Dentistry, Seoul National University Bundang Hospital, 172 Dolma-ro, Bundang-gu, Seongnam-si, Gyeonggi-do 13620 Republic of Korea; 2 Ieum Oral and Maxillofacial Surgery Dental Clinic, 45 Geumo-daero, Yesan-eup, Yesan-gun, Chungcheongnam-do 32428 Republic of Korea; 3 R&D Institute, Korea Tooth Bank, 56, Pyeongchang-gil, Jongno-gu Seoul, 03008 Republic of Korea; 4 Department of Dentistry and Dental Research Institute, School of Dentistry, Seoul National University, 101 Daehak-ro (Yeongeon-dong), Jongno-gu Seoul, 03080 Republic of Korea

**Keywords:** Dental implants, Demineralized dentin matrix, rhBMP-2 carrier

## Abstract

**Background:**

Traditionally, dental implants require a healing period of 4 to 9 months for osseointegration, with longer recovery times considered when bone grafting is needed. This retrospective study evaluates the clinical efficacy of demineralized dentin matrix (DDM) combined with recombinant human bone morphogenetic protein-2 (rhBMP-2) during dental implant placement to expedite the osseointegration period for early loading.

**Methods:**

Thirty patients (17 male, 13 female; mean age 55.0 ± 8.8 years) requiring bone grafts due to implant fixture exposure (more than four threads; ≥ 3.2 mm) were included, with a total of 96 implants placed. Implants were inserted using a two-stage protocol with DDM/rhBMP-2 grafts. Early loading was initiated at two months postoperatively in the mandible and three months in the maxilla. Clinical outcomes evaluated included primary and secondary stability (implant stability quotient values), healing period, bone width, and marginal bone level assessed via cone-beam computed tomography.

**Results:**

All implants successfully supported final prosthetics with a torque of 50Ncm, without any osseointegration failures. The average healing period was 69.6 days in the mandible and 90.5 days in the maxilla, with significantly higher secondary stability in the mandible (80.7 ± 6.7) compared to the maxilla (73.0 ± 9.2, *p* < 0.001). Histological analysis confirmed new bone formation and vascularization.

**Conclusion:**

DDM/rhBMP-2 grafting appears to significantly reduce the healing period, enabling early loading with stable and favorable clinical outcomes.

## Introduction

Autogenous bone is currently regarded as the optimal graft for bone induction because it possesses three critical properties required for bone formation: osteogenesis (the ability to form new bone), osteoconduction (the capacity to support the growth of new bone along its surface), and osteoinduction (the ability to induce bone formation) [1]. However, the use of autogenous bone grafts presents several limitations [2]. The graft must be harvested from the patient, which limits the available quantity of bone. Additionally, harvesting bone creates a secondary surgical site, leading to increased surgical time and blood loss. Morbidity at the donor site is a common and persistent issue. As a result, significant research in tissue engineering has been devoted to finding alternatives to autogenous bone grafts. Many of the bone graft substitutes undergo processing that denatures or removes proteins within the graft, leading to diminished or nonexistent osteoinductive properties. While xenografts are widely used in clinical practice and can achieve sufficient bone formation in favorable environments, they have the drawbacks of being difficult to monitor for bone formation at the terminal sites and being prone to infection.

Dentin shares chemical components with bone, including biological apatite (70%), collagen (18%), non-collagenous proteins (2%), and body fluids by weight. The dentin matrix is characterized by nano-sized dentinal tubules, typically ranging from 1 to 3 µm in diameter. These tubules play a key role in the release of intrinsic growth factors embedded within the matrix, as well as proteins that bind to hydroxyapatite. The density of dentinal tubules is approximately 18,000 to 21,000 per square millimeter [3]. The average porosity of these tubules is about 3.5%, which is notably lower than the 6.2% porosity found in natural human bones [3, 4]. The process of demineralizing the dentin matrix involves the extraction of inorganic salts while minimizing the leaching or denaturation of its organic components. As a result, a demineralized dentin matrix (DDM) emerges, characterized as a cell-free matrix composed of acid-insoluble, highly cross-linked type I collagen that contains matrix-binding proteins, including bone morphogenetic proteins (BMPs), within its microporous dentinal tubules. These osteoinductive elements and growth factors within DDM represent approximately 5% of the natural spectrum of growth factors, such as transforming growth factors, insulin-like growth factors, and BMPs. Additionally, BMPs derived from the tooth matrix exhibit similar biological activity as those sourced from bone tissue [5]. The porosity of dentinal tubules increases from 3%–6% to an average of 20%, while the uncollapsed freeze-dried interfibrillar space reaches approximately 50% [6]. The porous structure and collagen-rich matrix of DDM make it suitable as a carrier for recombinant human BMP-2 (rhBMP-2) [7, 8]. Studies have suggested synergy between endogenous BMPs and externally applied rhBMP-2. DDM combined with rhBMP-2 (AutoBT.BMP, Korea Tooth Bank, Seoul, Republic of Korea) results in more effective bone formation and osteocyte embedding compared to DDM alone [9]. Clinically, DDM has been recognized as a promising functional bone graft material in implant dentistry as well as an rhBMP-2 carrier [10].

The goal of dental implant surgery is to achieve osseointegration, where bone forms directly on the implant surface. Traditionally, a healing period of 3 months for the mandible and 5–6 months for the maxilla has been recommended to ensure successful osseointegration. In a two-stage procedure, implants are typically placed 4–9 months after autograft bone transplantation, with a longer healing period (≥ nine months) suggested for larger defects. This approach has allowed implants to be placed in relatively stable conditions [11]. Early loading of implants has been attempted when sufficient primary stability is achieved without the need for bone grafting [12]. BMPs, which are critical members of the highly conserved signaling proteins of the transforming growth factor-beta (TGF-β) superfamily, play a significant role in bone regeneration [13]. Several animal studies have shown that rhBMP-2 induces faster and greater initial bone formation and significantly increases the bone-to-implant contact ratio when used in conjunction with implant placement [14, 15]. Currently, rhBMP-2 is clinically utilized with carriers such as absorbable collagen sponge (ACS), biphasic calcium phosphate, β-tricalcium phosphate, hydroxyapatite, demineralized bone matrix, platelet-rich fibrin. Since rhBMP-2 needs to be released steadily at low concentrations within the body, a robust carrier that can sustain and bind to the rhBMP-2 is required [16]. Among these carrier ACS demonstrates a high binding capacity for rhBMP-2 and is widely used in oral and maxillofacial applications [16]. However, due to the physically instability of ACS, compression from surrounding soft tissues and fluids in the body can lead to the localized release of high doses of rhBMP-2, which may result in ectopic bone formation, swelling, erythematous, and even a potential risk of tumor [16]. Since DDM is mainly consist of type 1 collagen matrix, it has been suggested as a stable carrier for rhBMP-2, which could allow for the sequential and slow release of rhBMP-2 over a month [9]. The consistent release of rhBMP-2 during the first month may promote increased bone formation and potentially accelerate the timeline for bone healing, even at a reduced concentration of 0.2 mg/mL compared to the FDA-approved 1.5 mg/mL [9].

It is hypothesized that DDM combined with rhBMP-2 could enhance bone healing more effectively than autogenous bone alone. The authors propose that even when bone grafting is performed simultaneously with implant placement, DDM combined with rhBMP-2 may allow for earlier loading, potentially in less than three months. This study aimed to demonstrate clinical outcomes suggesting that grafting DDM incorporated with rhBMP-2 at the time of implant placement could reduce the healing period for the early loading.

## Materials and methods

All procedures in this study adhered to the ethical guidelines set by the institutional and national committees responsible for human experimentation, in accordance with the Helsinki Declaration of 1975, revised in 2008. Informed consent was obtained from all participants involved in the study. The study was approved by the Jeonbuk National University Hospital Institutional Review Board (IRB No. 2022-09-063). Through review of medical records from January 2020 to September 2022, patients were included according to the following inclusion criteria: (1) age over 20 years; (2) no history of uncontrolled systemic diseases or syndromes; (3) implant placement in the 5posterior mandible or maxilla within six weeks after extraction; (4) need for bone grafting due to implant fixture exposure (more than four threads; ≥ 3.2 mm); (5) a two-stage implant placement requiring simultaneous bone grafting using only autogenous demineralized dentin matrix incorporated with rhBMP-2; (6) a second surgery performed within 4 months of implant placement; (7) the presence of natural teeth, implants, or fixed prostheses in the opposing dentition; and (8) availability of cone-beam computed tomography (CBCT) imaging. Patients were excluded if they had undergone bone grafts mixed with other graft materials or membranes, or if there were missing records of follow-up or CBCT imaging.

Clinical outcomes, such as primary surgery stability (implant stability quotient; ISQ), healing period, secondary surgery stability (ISQ), and bone width measured on CBCT, were analyzed separately for implants placed in the maxilla and mandible. For the maxilla, the analysis was further divided into ridge augmentation and sinus grafting via lateral approach, where bone grafting was performed only at the fixture apex. Since sinus graft groups did not bone graft around the fixture neck area, the group was considered as a control group.

### Surgical procedure

Teeth that were deemed unsalvageable were extracted and sent to a manufacturer (Korea Tooth Bank, Seoul, Republic of Korea). The procurement, storage, processing, and packaging of teeth were individually handled in accordance with the Good Practice Guidelines for Tooth Handling Institutions, as stipulated by the Korea Administration of Health and Welfare [17]. The processing method for the demineralized dentin matrix (DDM) includes refrigerating the teeth in 70% ethyl alcohol, followed by rinsing and removing any attached soft tissue and pulp using a retrograde technique. The dentin is then ground into particles (300–800 μm), followed by defatting and demineralization with 0.6 N HCl, incorporating a viral inactivation procedure as described in Patent EP 2601982, resulting the volume ranged from 0.4 to 1.0 cc [18]. Subsequently, rhBMP-2 is applied to the DDM powder at a concentration of 0.2 mg/mL (Cowellmedi, Seoul, Republic of Korea).

**Fig. 1:**
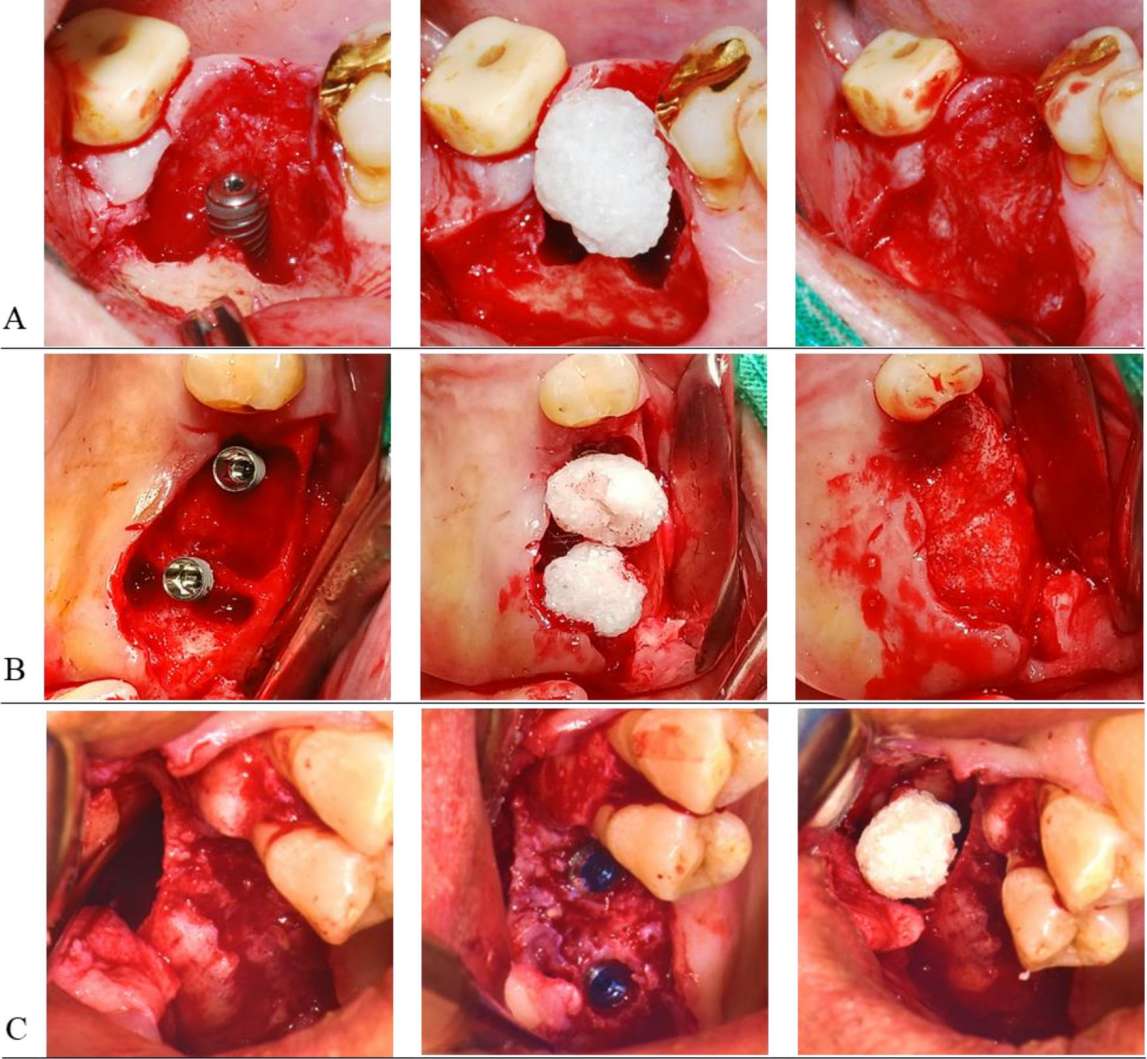
Representative surgical procedure of AutoBT.BMP graft according to the surgery types. **A** Mandibular ridge augmenation for exposed implant fixture. **B** Maxillary ridge augmenation for exposed implant fixture. **C** Maxillary sinus graft for the apex of implant fixture

Implant placement and bone grafting at the extraction site were performed within 4–6 weeks after extraction. If other teeth required extraction at sites other than the implant surgery site, AutoBT.BMP was prepared in advance, and surgery was performed immediately after extraction. The implant was placed, and a cover screw was connected when a minimum initial fixation force of 30Ncm was achieved [19]. AutoBT.BMP was grafted around the exposed implant threads, and primary closure was achieved by using 4–0 vicryl (Ethicon, Johnson & Johnson International, New Jersey) without the use of a membrane or other bone substitute (Fig. 1).

All patients underwent single CBCT (Sirona scanner, Dentsply Sirona, Charlotte, North Carolina; 85 kV and 6.5 mA; voxel size of 160 μm; a scanning time of 14 s) imaging immediately after surgery. Routine postoperative care included the administration of antibiotics and anti-inflammatory analgesics for five days, with sutures being removed after one week.

Secondary surgery was performed on average after 2 months for the mandible and 3 months for the maxilla, with loading occurring within three weeks of secondary surgery. During the second surgery, if bone tissue formed above the cover screw could be collected, a biopsy was performed with the patient's consent. The implant stability was evaluated during the second operation using the Osstell Mentor device (Osstell, Gothenburg, Sweden) as ISQ value [20]. CBCT imaging and histological examination of the bone tissue above the implant cover screw were performed during the secondary surgery for consenting patients.

### Measurement of bone graft using cone-beam computed tomography

**Fig. 2:**
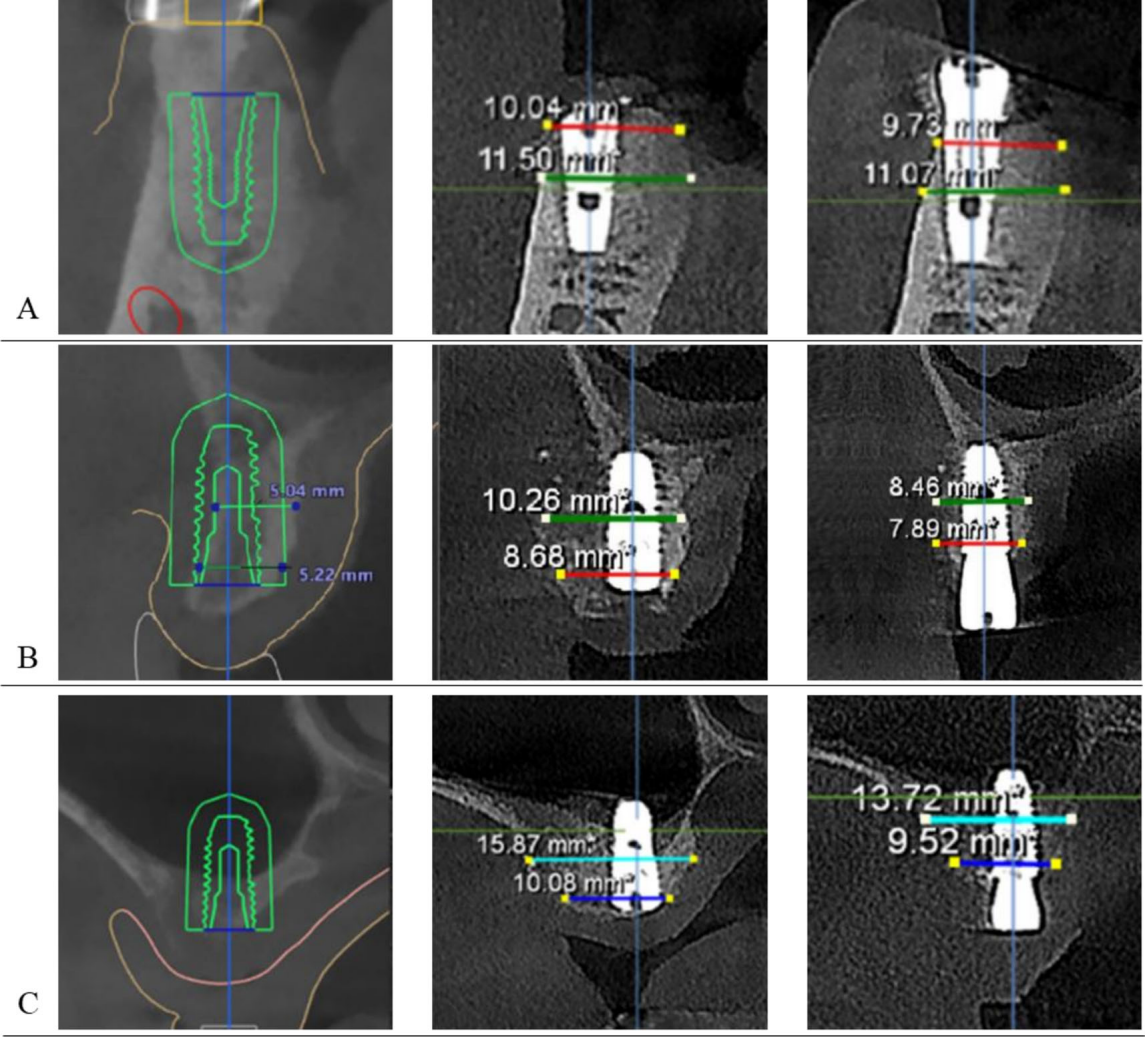
Measurement of horizontal ridge width at each time points according to the surgery types. **A** Mandibular ridge augmenation for exposed implant fixture. **B** Maxillary ridge augmenation for exposed implant fixture. **C** Maxillary sinus graft for the apex of implant fixture

Using linear measurement tools on CBCT, two examiners (J.H. Lim and J.A. Lim) independently measured vertical marginal bone height and horizontal bone width, and the results were averaged. The marginal bone height around the implant was measured along the center of the implant in a cross-sectional slice, from the crest of the buccal and lingual marginal bone to the base of the implant. In the cross-sectional view, a vertical reference line was established from the radiolucent center of the cover screw to the midpoint of the fixture. A horizontal reference line was then drawn perpendicular to this vertical line at the level of the marginal bone crest. Measurement of the marginal bone was taken using these reference lines, relative to both the marginal crest and the apex of the dental implant [21]. At the same reference line, horizontal bone width was measured at 1 mm and 5 mm below the highest point of the fixture thread. Measurements were taken immediately after the bone graft and at the time of the second surgery (Fig. 2).

### Histological observation and histomorphometry analysis

Specimens were fixed in 10% neutral-buffered formalin and then demineralized using 10% formic acid. Longitudinal sections, 5–8 μm thick, were prepared from the central region of the specimens using a microtome. The sections were then stained with hematoxylin and eosin (H&E) and scanned using a Panoramic 250 Flash III scanner (3DHISTECH, Budapest, Hungary). Following this, the samples were fixed in 10% buffered formalin and decalcified with 10% formic acid. Histological evaluation involved H&E staining as well as immunohistochemical staining for osteocalcin and BMP-2 [22, 23]. The scanned slides were observed using slide-viewing software (Case Viewer ver. 2.1, 3DHISTECH, Budapest, Hungary).

### Statistical analysis

The parametric assumptions of the data were evaluated using the Kolmogorov–Smirnov test. An independent samples t-test was conducted to compare the jaw and types of graft (ridge augmentation and sinus graft) in the maxilla. Statistical analysis was performed using SPSS 25.0 for Windows (SPSS Inc., Chicago, IL, USA). Data were presented as the mean ± standard deviation.

## Results

### Clinical results

The study included 30 participants (17 males and 13 females, with a mead age 55.0 ± 8.8 years). A total of 96 implants (46 in the mandible and 50 in the maxilla) were placed. The implants had sandblasted, acid-etched surfaces with internal hex connections (TSIII SA, Osstem, Seoul, Republic of Korea; SuperLine, Dentium, Suwon, Republic of Korea). A two-stage protocol was followed, with an initial insertion torque of > 35 Ncm, and simultaneous grafting with AutoBT.BMP. After the average healing period was 82.4 ± 19.4 days, the implant stability quotient (ISQ) at the second surgery averaged 75.5 ± 9.5 (Table 1) [24]. There were no cases of implant osseointegration failure, and all final prostheses were of the SCPR type and were secured with a torque of over 50Ncm.

**Table 1:** Demographic and clinical information of the included patients

Variables	
Age (years)	55.0 ± 8.8
Male:Female (n)	53:43
Mandible:Maxilla (n)	46:50
TSIII SA:SuperLine (n)	47:49
Healing period (days)	82.4 ± 19.4
ISQ value	75.5 ± 9.5

According to the jaw, 46 and 50 implants were placed on the mandible and maxilla, respectively (Table 2). There were no statistically significant differences in gender or age. The healing period was on average 20.9 days shorter in the mandible (*p* < 0.001), and the implant stability was lower in the maxilla (71.7 ± 9.4) compared to the mandible (80.9 ± 6.5) (*p* < 0.001).

**Table 2:** Comparison of clinical variables in the implants according to the jaw

Variables	Maxilla (n = 50)	Mandible (n = 46)	*p*
Male:Female (n)	30:20	23:23	0.429*
Age (years)	54.5 ± 7.9	55.5 ± 9.6	0.556**
Healing period (days)	90.5 ± 17.7	69.6 ± 16.2	< 0.001**
ISQ value	71.7 ± 9.4	80.9 ± 6.5	< 0.001**

*Pearson's Chi-square test

**Independent t-test

Regarding the maxilla (Table 3), AutoBT.BMP was grafted for ridge augmentation on 36 implants and for sinus grafting on 14 implants without bone graft on the marginal bone. Age, gender, and recovery period were similar, but the implant stability was lower in the group that had only the sinus graft. (*p* = 0.003).

**Table 3:** Comparison of clinical variables in the implants on maxilla between the bone graft types

Variables	Ridge augmentation (n = 36)	Sinus graft (n = 14)	*p*
Male:Female (n)	23:13	7:7	0.419*
Age (years)	53.0 ± 8.1	57.5 ± 6.8	0.056**
Healing period (days)	88.1 ± 18.2	93.2 ± 13.9	0.302**
ISQ value	74.1 ± 9.2	66.0 ± 7.4	0.003**

*Pearson's Chi-square test

**Independent t-test

### Measurement of marginal bone change using cone-beam computed tomography

Immediately after bone graft surgery and secondary surgery, CBCT was performed on a total of 33 maxillary implants (19 men, 14 women, average age 53.8 ± 8.4 years). Among them, 23 implants underwent ridge augmentation due to the exposure of three or more threads of the fixture, and 10 implants underwent sinus grafting without fixture exposure. There was no difference in the implant marginal bone level and ridge horizontal width between the group that underwent ridge augmentation using AutoBT.BMP and the group that did not graft the marginal bone (sinus graft) (Table 4).

**Table 4:** Comparison of clinical information of maxillary implants between different bone graft types, using CBCT at pre-surgery, immediately post-surgery, and at the time of secondary surgery, considering only implants present at these time points

Variables	Ridge augmentation (n = 23)	Sinus graft (n = 10)	*p*
Male:Female	14:9	5:5	0.576*
Age	52.3 ± 8.5	57.3 ± 7.5	0.120**
Healing period (days)	76.7 ± 17.3	87.0 ± 18.1	0.145**
Ridge width at 1 mm below the fixture at the immediately post-operation (mm)	10.06 ± 2.10	10.55 ± 2.72	0.578**
Ridge width at 5 mm below the fixture at the immediately after the surgery (mm)	11.66 ± 2.44	13.31 ± 2.10	0.073**
Marginal bone level at immediately after the surgery (mm)	9.12 ± 2.67	10.87 ± 2.77	0.172**
Ridge width at 1 mm below the fixture at the second surgery (mm)	9.07 ± 2.10	9.19 ± 2.21	0.889**
Ridge width at 5 mm below the fixture at the second surgery (mm)	10.99 ± 2.49	12.38 ± 1.99	0.128**
Marginal bone level at the second surgery (mm)	9.29 ± 2.17	9.27 ± 1.54	0.986**
Changes of Ridge width at 1 mm below the fixture (mm)	0.85 ± 1.19	1.28 ± 1.10	0.355**
Changes of Ridge width at 5 mm below the fixture (mm)	0.71 ± 0.92	0.96 ± 0.86	0.499**
Changes of marginal bone level (mm)	0.05 ± 0.81	0.94 ± 1.62	0.147**

*Pearson's Chi-square test

**Independent t-test

### Histological results

**Fig. 3:**
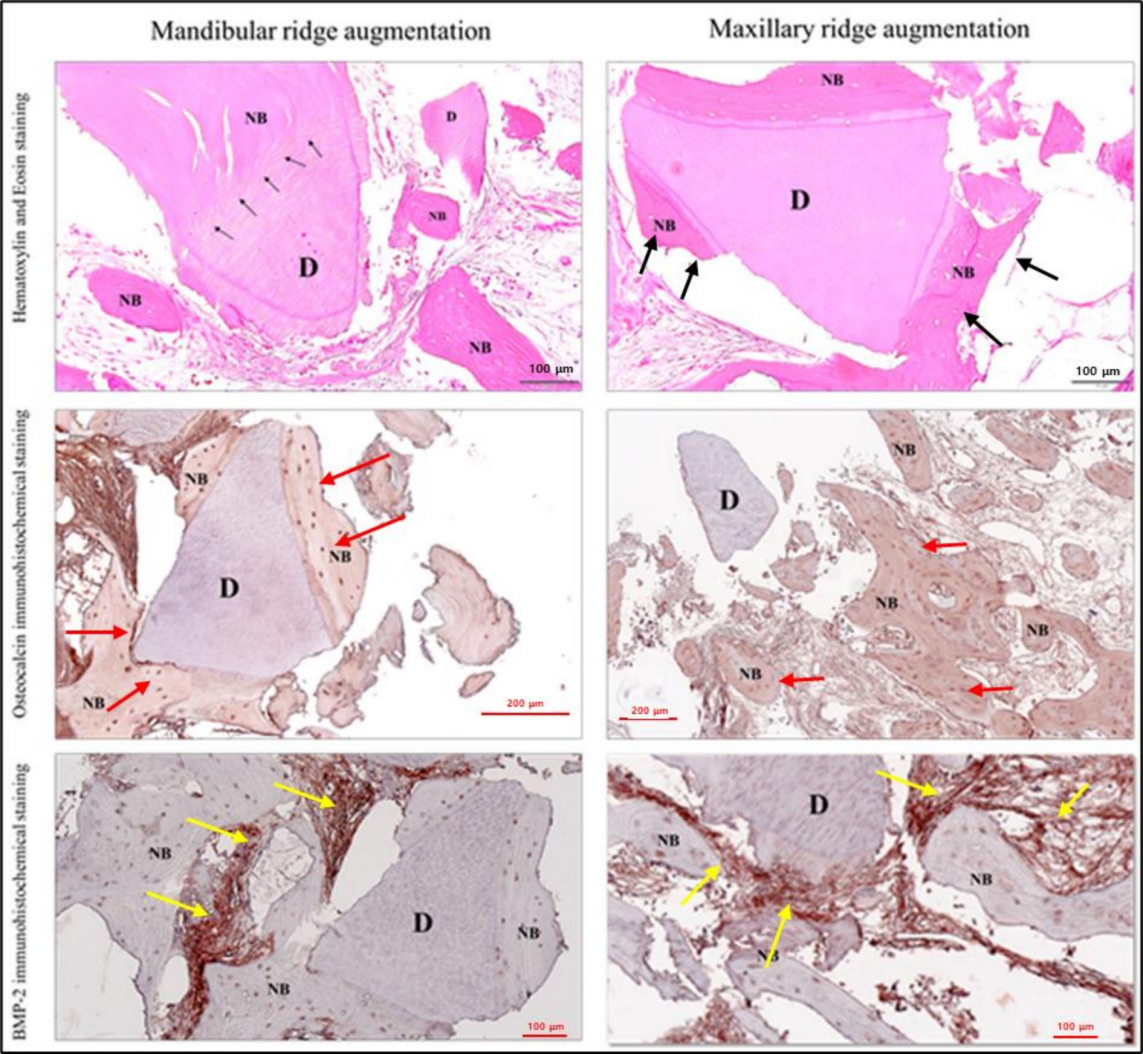
Histological results of ridge augmentation from the mandible at 70 days and from the maxilla at 101 days postoperatively. (D: DDM particle; NB. New bone) **A** Histological images of AutoBT.BMP graft in mandible. High magnification of DDM on gingival side showed osteoconductive and osteoinductive bone formation. There are loose fibrous connective tissues between DDM particle and newly formed bone. The border between DDM and new bone can not be detected that is like aponeurosis (black arrow). **B** Histological images of AutoBT.BMP graft in maxillae. High magnification of lower root of specimen showed osteoinductive bone formation of DDM. Bone marrow like structures are developed. **C** OCN (Osteocalcin)‐staining images of DDM/rhBMP-2 composites in maxilla. OCN positive cells (red arrow) appeared around osteocytes in newly formed bone as well as between the new bone and DDM particle. **D** OCN‐staining images of AutoBT.BMP graft in mandible. OCN positive cells (red arrow) appeared around osteocytes in newly formed bone. E. BMP-2 staining images of AutoBT.BMP graft in maxilla. There were no specific identification of BMP-2 in dentin and newly formed bone. Fibrous tissues between DDM particle and new bone showed abundant BMP-2 positive cells (yellow arrow). F. BMP-2 staining images of AutoBT.BMP graft in mandible. There were no specific identification of BMP-2 in dentin and newly formed bone. Fibrous tissues between DDM particle and new bone showed abundant BMP-2 positive cells (yellow arrow)

Two patients provided consent for histological examination. Tissue samples were obtained from the mandible at 70 days and from the maxilla at 101 days during secondary surgery, specifically from the cover screw area. Histological analysis of the grafted AutoBT.BMP revealed osteoinductive bone healing outcomes in the both of mandible and maxilla (Fig. 3). In the mandible at 70 days (Fig. 3A), detailed examination of DDM on the gingival side showed both osteoconductive and osteoinductive bone formation, with loose fibrous connective tissues between DDM particles and newly formed bone. The boundary between DDM and new bone appeared indistinct, resembling aponeurosis (black arrow). In the maxilla at 101 days (Fig. 3B), high magnification of the lower root exhibited osteoinductive bone formation of DDM, accompanied by the development of structures similar to bone marrow.

Staining with Osteocalcin (OCN) further highlighted these observations: in the maxilla (Fig. 3C), OCN-positive cells (red arrow) were evident around osteocytes within newly formed bone and between the new bone and DDM particles. In the mandible (Fig. 3D), OCN-positive cells (red arrow) were observed around osteocytes in newly formed bone.

BMP-2 staining images revealed significant findings: in the maxilla (Fig. 3E), BMP-2 was not specifically identified in dentin or newly formed bone, but fibrous tissues between DDM particles and new bone exhibited abundant BMP-2-positive cells (yellow arrow), consistent with observations in the mandible (Fig. 3F).

## Discussion

The author hypothesized that AutoBT.BMP would effectively demonstrate the osteoinductive potential of demineralized dentin matrix (DDM) as a bone graft material by leveraging the synergistic effects of its nanoporous structure and collagen-rich matrix as a carrier for rhBMP-2. The study's findings support this hypothesis, showing that AutoBT.BMP facilitated successful osseointegration across all implants, with no failures observed, and maintained satisfactory bone volume. Histological analysis further indicated that DDM could serve as an effective alternative to autogenous bone. The osteoinductive capability, which promotes bone formation by directly inducing osteoblast differentiation through vascular formation and mesenchymal cell recruitment at the graft site, is anticipated to enhance osseointegration around the implant fixture, potentially shortening the traditional bone healing period. The patients in this study achieved an initial fixation strength of 30Ncm and underwent secondary surgery after an average of 2 months for the mandible and 3 months for the maxilla to enable early loading. During secondary surgery, an average fixation strength of 75.5 ISQ was recorded, and all final prostheses were successfully mounted using a torque of over 50Ncm, with no osseointegration failures.

BMP is a naturally occurring multifunctional protein initially discovered for its bone-inducing capabilities as a secreted cytokine. BMP signaling is mediated through serine/threonine kinase receptors [25]. It can activate p38 mitogen-activated protein kinase (MAPK), extracellular signal-regulated kinase (ERK), and c-Jun N-terminal kinase (JNK) signaling pathways, stimulating the expression of key osteogenic transcription factors such as runt-related transcription factor 2 (Runx2), distal-less homeobox 5 (Dlx5), and osterix (Osx). Runx2 plays a crucial role in osteogenesis [26, 27]. At the cellular level, BMP functions as a ligand for receptors on various cells, including osteoblasts, osteoclasts, adipose stem cells, mesenchymal stem cells, and tendon fibroblasts, promoting their differentiation and proliferation [28, 29]. Bone marrow stromal cells are regulated by the Runx2 and Osx genes, with Runx2 being the primary driver throughout the differentiation process. The BMP-2 signaling pathway directly influences Runx2 expression, thereby enhancing osteoblast differentiation and regulating bone formation [24, 30–32]. As a result, BMP-2 significantly promotes the formation of mineralized nodules, osteogenic differentiation, and bone healing [33–36].

In 2002, the FDA approved rhBMP-2 for clinical use in spinal fusion, treatment of open or nonunion fractures, and maxillofacial bone augmentation, delivered via a collagen sponge [37]. Several studies have reported that the use of rhBMP-2 in the field of oral and maxillofacial surgery increases the amount of bone formation [38, 39]. Absorbable collagen sponges are employed as rhBMP-2 carriers for maxillary sinus grafts and tooth extractions, with FDA approval at a concentration of 1.5 mg/mL [40, 41]. However, common adverse events include oral pain, edema, and erythema, largely due to burst release from the carrier's mechanical instability in a physiological environment [42]. Effective carrier scaffolds must maintain a BMP concentration at the local site high enough to support healing while avoiding adverse effects. In comparison to other carriers such as absorbable collagen sponge (ACS) and β-tricalcium phosphate (β-TCP), demineralized dentin matrix (DDM) offers distinct advantages. While ACS has a high binding capacity for rhBMP-2, its burst release under physiological pressure can lead to adverse effects such as ectopic bone formation and inflammation. DDM, with its nano-porous structure and collagen-rich matrix, provides a more stable release profile and better integration with surrounding bone tissue. In 2019, it was proposed that the hypothesized release profile of DDM combined with rhBMP-2 includes both physically adsorbed and modified, as well as physically entrapped rhBMP-2, sequentially released from the DDM surface during the initial implantation phase over 36 days, including the latest emerging endogenous BMPs within the dentin.[9] Thus, DDM/rhBMP-2 grafts can maintain high BMP activity for at least one month post-implantation, leading to significant early bone formation and a reduced bone healing period. Although dentin is a collagen matrix, it has greater density and physical stability compared to collagen sponges. When grafted around implants, DDM/rhBMP-2 can function effectively for up to a month without burst release, potentially enabling faster osseointegration.

Some animal studies have reported that rhBMP-2 reduces bone healing time and enhances implant osseointegration [14, 15]. However, few studies have examined the application of rhBMP-2 to the exposed implant area for early loading. When bone grafting is necessary for bone defects in humans, primary closure of deficient soft tissue can cause compression, raising concerns about the rapid release of rhBMP-2 under physiologic pressure [43]. In previous studies using DDM as a carrier for rhBMP-2, there were no complications from rapid release, even in challenging periodontal pocket grafting where soft tiszsue coverage is challenging [44]. Therefore, DDM/rhBMP-2 grafts around implants could stably facilitate early osseointegration and subsequent loading. In this study, early loading achieved a stability of 50Ncm in all cases, with an average ISQ of 75.5 and at least 68.0 in sinus graft cases, indicating that loading was feasible [45, 46]. The ISQ value measures the lateral stiffness of the bone-implant interface and the rigidity of the surrounding bone. Implant stability tends to decrease during the initial healing phase due to bone resorption at the healing site [47]. According to Nappo et al. in 2019, in cases with low bone density in the maxilla, the ISQ value tends to decrease during the osseointegration process, even if the healing period is extended [48]. Interestingly, in our study, after a three-month healing period, the ISQ value did not increase in the maxilla (Table 2). Due to more extensive bone remodeling in the maxilla, maxillary implants can show greater ISQ increases with function [49, 50]. Since implant stability is closely related to the condition of the marginal bone around the implant fixture [51], the ridge augmentation group was compared, which underwent marginal bone grafting in the maxilla, with the sinus graft group where host bone was present (Table 3 and 4). The ISQ values and CBCT findings of bone resorption in the group that received grafting at the fixture top were not inferior to the response seen in host bone. Additionally, histological analysis of bone tissue obtained from the implant cover screw (Fig. 3), an area least likely to exhibit bone formation, showed new bone formation and vascularization at one month in the mandible and at one and three months in the maxilla, suggesting significant bone formation around the implant.

Shortening the overall healing period to achieve efficient clinical outcomes is a goal for many clinicians. Evaluating bone healing after bone grafting typically requires histological confirmation, often necessitating studies where trephine burs are used to assess bone healing during implant placement. In 2023, platelet–rich fibrin and demineralized bovine bone mineral were used, and results showed an implant stability quotient (ISQ) of over 60 after 4 months, with histomorphometric analysis demonstrating greater bone formation and faster bone healing compared to controls [52]. However, they involved a four-walled defect, which is conducive to bone formation in the maxillary sinus, and despite setting a long healing period of 4 months for the experimental group and 8 months for the control group. Few studies have attempted to shorten the bone healing period to less than 4 months, especially in challenging cases involving simultaneous bone grafting and implant placement in humans. In this study, without a control group and unable to directly assess the extent of osseointegration of the actual implants, it is difficult to conclusively determine if DDM/rhBMP-2 significantly shortened the healing period. Nevertheless, this study involved 1–2 walled defects, which are more challenging for bone formation, and yet no implant failures were observed despite a short healing period of 2 months for the mandible and 3 months for the maxilla. Moreover, clear new bone formation was observed in histological results (Fig. 3) in areas least likely for bone formation, such as above the cover screw, supporting the clinical validity of a shorter healing period.

This study is the first to explore early loading in patients undergoing bone grafting with DDM/rhBMP-2 at sites with exposed implants. If further validated, these findings could revolutionize traditional implant dentistry protocols, which typically require a 5–6 months healing period post-bone grafting or simultaneous implant placement before loading. While the primary focus of this study is on dental implant surgery, the findings have broader implications for other fields of bone regeneration, particularly in orthopedics. The combination of DDM and rhBMP-2 could be applied to the treatment of long bone fractures, non-union fractures, and osteonecrosis, where rapid bone regeneration and vascularization are critical. Additionally, the ability of DDM/rhBMP-2 to support early loading in dental implants suggests its potential use in orthopedic bone augmentation and reconstruction procedures. However, as a retrospective study, it has several limitations. The lack of a control group, the inability to measure ISQ during primary surgery, and the absence of CBCT imaging during secondary surgery in all cases are notable. Moreover, since this was a study involving human subjects, it was not possible to evaluate the bone-implant contact ratio to precisely determine osseointegration at the time of loading. Future animal studies and well-designed prospective studies are necessary.

This study suggests that incorporating DDM with rhBMP-2 during implant placement shows promise in reducing the healing period, potentially allowing for earlier loading. However, as a retrospective study, it has several limitations. One of the main limitations of this study is the restricted histological sample size, with only two patients consenting to provide biopsy specimens for detailed analysis. This limited number of histological samples restricts the robustness and generalizability of our findings regarding the osteoinductive effects of the DDM/rhBMP-2 graft. Notably, the absence of a control group without rhBMP-2 limits the strength of the conclusions. Further well-designed studies with appropriate control groups are necessary to confirm these findings and establish the comparative effectiveness of this approach in reducing the healing period for early implant loading.

## Data Availability

All data generated or analyzed are included in this article. Further inquiries can be directed to the corresponding author.
